# Early Influences of Microbiota on White Matter Development in Germ-Free Piglets

**DOI:** 10.3389/fncel.2021.807170

**Published:** 2021-12-27

**Authors:** Sadia Ahmed, Sierrah D. Travis, Francisca V. Díaz-Bahamonde, Demisha D. L. Porter, Sara N. Henry, Julia Mykins, Aditya Ravipati, Aryn Booker, Jing Ju, Hanzhang Ding, Ashwin K. Ramesh, Alicia M. Pickrell, Maosen Wang, Stephen LaConte, Brittany R. Howell, Lijuan Yuan, Paul D. Morton

**Affiliations:** ^1^Graduate Studies in Biomedical and Veterinary Sciences, Virginia-Maryland College of Veterinary Medicine, Virginia Tech, Blacksburg, VA, United States; ^2^Department of Biomedical Sciences and Pathobiology, Virginia-Maryland College of Veterinary Medicine, Virginia Tech, Blacksburg, VA, United States; ^3^Virginia Tech Graduate Program in Translational Biology, Medicine and Health, Virginia Tech, Roanoke, VA, United States; ^4^Molecular Microbiology and Immunology, University of Missouri, Columbia, MO, United States; ^5^School of Neuroscience, Virginia Tech, Blacksburg, VA, United States; ^6^Fralin Biomedical Research Institute at Virginia Tech Carilion (VTC), Virginia Tech, Roanoke, VA, United States; ^7^Biomedical Engineering and Mechanics, Virginia Tech, Blacksburg, VA, United States; ^8^Department of Human Development and Family Science, Virginia Tech, Roanoke, VA, United States

**Keywords:** microbiota, brain, white matter, oligodendrocyte, myelin, germ-free, swine

## Abstract

Abnormalities in the prefrontal cortex (PFC), as well as the underlying white matter (WM) tracts, lie at the intersection of many neurodevelopmental disorders. The influence of microorganisms on brain development has recently been brought into the clinical and research spotlight as alterations in commensal microbiota are implicated in such disorders, including autism spectrum disorders, schizophrenia, depression, and anxiety *via* the gut-brain axis. In addition, gut dysbiosis is common in preterm birth patients who often display diffuse WM injury and delayed WM maturation in critical tracts including those within the PFC and corpus callosum. Microbial colonization of the gut aligns with ongoing postnatal processes of oligodendrogenesis and the peak of brain myelination in humans; however, the influence of microbiota on gyral WM development remains elusive. Here, we develop and validate a neonatal germ-free swine model to address these issues, as piglets share key similarities in WM volume, developmental trajectories, and distribution to humans. We find significant region-specific reductions, and sexually dimorphic trends, in WM volume, oligodendrogenesis, and mature oligodendrocyte numbers in germ-free piglets during a key postnatal epoch of myelination. Our findings indicate that microbiota plays a critical role in promoting WM development during early life when the brain is vulnerable to environmental insults that can result in an array of disabilities manifesting later in life.

## Introduction

Though the brain serves as the master regulator of the body, it is heavily influenced by other systems with hubs located in distant compartments. The gut-brain axis (GBA) is one example of such crosstalk inclusive of a bidirectional network fostering communication between the central nervous system (CNS), autonomic nervous system (ANS), enteric nervous system (ENS), and hypothalamic pituitary adrenal (HPA) axis (Carabotti et al., 2015). Studies providing evidence of the therapeutic importance of the GBA were historically seeded by observations of psychological stress as a comorbidity in human patients with inflammatory bowel disease (Mawdsley and Rampton, 2005; Cámara et al., 2009; Cryan and O’Mahony, 2011; Kennedy et al., 2012). Because the gut is a major source of microorganisms (i.e., bacteria, viruses, fungi, and protozoa), along with their collective genetic materials (i.e., microbiome), alterations in diversity or composition of gut microbiota can impact the brain; thus resulting in the modern nomenclature of gut microbiota-brain axis or brain-gut microbiome axis defining a reciprocal relationship (Martin et al., 2018; Morais et al., 2021).

There is an abundance of clinical evidence suggesting that dysfunction of the GBA is a common culprit underlying a wide spectrum of neurodevelopmental disorders. For example, intestinal complications (de Magistris et al., 2010), changes in microbial composition (Louis, 2012), and food allergies (de Theije et al., 2014) have been documented in pediatric patients with autism spectrum disorders (ASD). Dietary immunomodulation issues (Verlaet et al., 2014), allergic sensitization (de Theije et al., 2014), and reductions in microbiome alpha diversity are seen in young patients with attention deficit hyperactivity disorder (ADHD; Prehn-Kristensen et al., 2018). Gut dysbiosis preceding immune imbalances has also been shown in human schizophrenia cases (Severance et al., 2013, 2016).

Infants born prematurely or with congenital complications, often spend a long time in microorganism-free isolators and/or are given antibiotics to prevent infection. In addition, gut dysbiosis is common in preterm birth, representing 10% of all live births in the US, where these patients often display diffuse WM injury and delayed WM maturation concurrent with the onset of neurodevelopmental disorders—including alterations in PFC and interconnecting WM tracts from the genu of the corpus callosum (CC) in adolescence (Botting et al., 1997; Anderson and Doyle, 2003, 2004; Dyet et al., 2006; Thompson et al., 2007; Narberhaus et al., 2008; Delobel-Ayoub et al., 2009; Ball et al., 2012; de Kieviet et al., 2012; Claud et al., 2013; Molnar and Rutherford, 2013; Back, 2014; Back and Miller, 2014; Brooks et al., 2014; Nosarti et al., 2014; Taft et al., 2014). Collectively, there is a strong link between aberrations in healthy commensal microbiota early in life and subsequent mental illnesses manifesting later during maturation.

Numerous studies in rodent models have established a functional relationship between the gut microbiome and brain development, including effects on neurochemical, hormonal, and behavioral attributes of their hosts (Sampson and Mazmanian, 2015; Vuong et al., 2017); however, a majority of these studies provide outcome measures during adolescence or adulthood, leaving a dearth of knowledge regarding the impact on early brain development. Furthermore, little is known regarding the influence of gut microbiota on white matter (WM) development in any species. Currently, our knowledge is limited to contradictory findings, as hypermyelination and hypomyelination have been reported in two separate germ-free (GF) mouse studies (Hoban et al., 2016; Lu et al., 2018). Although these studies assessed the functional and behavioral outcomes later in life due to the absence of gut microbiota, they did not evaluate mechanistic changes during the protracted and complicated process of WM development in early life.

WM comprises ~50% percent of adult human brain volume (Sampaio-Baptista and Johansen-Berg, 2017) and is essential for sensory, motor, cognitive, and social skills (O’Muircheartaigh et al., 2014). WM development is a protracted and highly orchestrated process involving the production, migration, and maturation of oligodendrocytes followed by myelination of target axons. Unlike rodents, where myelination in the brain begins postnatally, the onset of myelination in human brains occurs as early as the 2nd trimester; and myelination of axons generally proceeds asymmetrically from inferior to superior and posterior to anterior through adolescence (Semple et al., 2013). Significant perturbations during WM maturation periods can contribute to neurological and psychiatric disorders later in life such as ASD, schizophrenia, depression, and ADHD (Fields, 2008). Importantly these disorders are also commonly associated with gut dysbiosis. Since microbial colonization of the gut at birth aligns with the ongoing process of oligodendrogenesis and the peak of brain myelination, understanding the impact of the gut microbiome on any aspect of WM development in an experimentally tractable species similar to humans is important.

Studies have validated the gut-brain-behavior interaction in domestic pigs under psychosocial stress conditions (chronic stress based on social isolation) showing the relationship between gut physiology (increased permeability, lower number of goblet cells, etc.) and microbiota activity (lower levels of SCFAs) combined with altered peripheral and brain immunological status that impact Hypothalamic-Pituitary-Adrenal (HPA) axis to depressive behavior (Menneson et al., 2019, 2020). Another study showed that pigs supplemented with *Lactobacillus* probiotics in early life exhibit an altered anxiety-like state, e.g., prevent increasing vigilance behavior in response to an auditory threat (Verbeek et al., 2021). These studies are among those that have paved the way to utilize piglets as a model for neurological and psychiatric diseases.

In this study, we utilized a germ-free piglet model to assess the impact of microbiota on WM development during early postnatal life. Unlike mice where WM represents ~15% brain volume (Ventura-Antunes et al., 2013), pigs have ~50% (Stinnett et al., 2017) which is comparable to humans. In addition, pigs are endowed with a gyrencephalic neocortex and gyral WM similar to humans (Sjöstedt et al., 2020). Importantly, the onset of myelination occurs *in utero* in swine which better recapitulates the human scenario. In addition to well-known parallels in brain structure, developmental trajectories, anatomy, physiology, and the immune system, pigs possess similar gastrointestinal tracts as their human counterpart (Litten-Brown et al., 2010). Similar bacterial diversity patterns were reported in humans and domestic swine with *Bacteriodetes* and *Firmacutes* genera predominating (Lamendella et al., 2011). Our findings using a multipronged approach including *ex vivo* MRI, cellular analyses, and biochemical methods demonstrate a significant impact of microbiota on WM volume and oligodendrocyte numbers within two key WM tracts of the developing piglet brain.

## Materials and Methods

### Animals

Male and female domestic pigs (*Sus scrofa*, Landrace × Yorkshire crossbreed) were used for all experiments. A total of 44 piglets were used in this study: necropsy (GF, *n* = 4) and serum component assessments (C, *n* = 7; GF, *n* = 4) at P42, MRI (C, *n* = 4; GF, *n* = 6), western blots (C, *n* = 3; GF, *n* = 3), histology (C, *n* = 4; GF, *n* = 3) at P16, and histology (*n* = 3–6) at P0. With the exception of necropsy and serum components, each group of pigs was used for the aforementioned assays with no overlap. Owing to animal housing restrictions, piglets can be reared in these isolators for up to 6 weeks, therefore, histopathological and serum component assessments were made at P42 following the longest exposure to GF conditions. Pigs were procured *via* Oak Hill Genetics, IL, and the Swine Agricultural Research and Extension Center at Virginia Tech. All experiments were conducted in accordance with the NIH Guide for the Care and Use of Laboratory Animals and carried out under the approval of the Virginia Tech Institutional Animal Care and Use Committee.

### Germ-Free Paradigm

Control and Germ-free (GF) piglets were born from different parents. GF piglets were derived *via* hysterectomy from near-term sows 1 day prior to term delivery as previously described in detail. To prevent iron deficiencies, 150 mg of iron dextran was administered intramuscularly immediately following birth. Piglets were housed in sterile isolators with dimensions (24" × 42"× 24") designed to accommodate up to four piglets. The piglets were able to hear and smell other animals housed in the same isolator and one toy was provided per chamber. Animals were kept on a 12:12 h light: dark cycle. The room temperature is maintained between 93 and 95°F initially and then decreased 2°F/week for the remainder of the study. Pigs were fed a sterile commercial diet (Hershey’s UHT milk) three times a day; through individual feeders provided for each animal to ensure a controlled diet that increased in volume to accommodate growth. Piglets were closely monitored throughout the day for any clinical signs of concern by the Teaching and Research Animal Care Support Unit (TRACSS) and/or an on-site/call veterinarian. Animals were weighed weekly and GF status was confirmed biweekly by sterility tests of rectal swabs for aerobic and anaerobic microbes using blood agar and thioglycollate media, respectively; additionally, the isolators were swabbed and tested in the same manner. All items (e.g., weighing instruments, swabs, toys, etc.) were sterilized with Spor-Klenz^®^ prior to introduction to the isolators. Healthy, GF animals were euthanized in accordance with AVMA Guidelines for the Euthanasia of Animals for the following experimental procedures. Control piglets were delivered vaginally and raised in a conventional agricultural environment. P0 piglets were euthanized shortly following birth *via* hysterectomy.

### Serum and Tissue Processing

Prior to euthanasia, animals were weighed. Blood was drawn from the heart, incubated in BD Vacutainer^®^ serum blood collection tubes for 1 h at room temperarture (RT), and subsequently stored on ice until centrifugation at 1,000× *g* for 10 min to isolate serum which was immediately delivered to Virginia Tech Animal Laboratory Services (ViTALS) for quantification of cortisol, cholesterol, and glucose levels. Owing to the diurnal nature of cortisol, bood was collected from all animals within each cohort within a 3-h window (9 AM-12 PM).

Quickly following euthanasia, brains were removed from the skull, weighed, and cut at 5 mm intervals on a custom-designed brain mold, and fixed at 4°C in 4% paraformaldehyde (0.1 M PBS) for 72 h. After fixation, tissue slabs were cryoprotected at 4°C in a sucrose gradient of 20% and 30% (0.1 M PBS) until sunken. Tissue was embedded in OCT compound (Tissue-Plus #4585) and stored at −80°C for subsequent serial sectioning on a Thermo Scientific CryoStar NX50 cryostat. Coronal tissue sections (50 μm) were collected for immunohistochemical procedures. For tissue used in H&E and DAB staining, 5 mm tissue blocks were incubated in 10% formalin for 72 h, embedded in paraffin, and cut on a microtome at 5 μm thickness. Histopathological assessments were performed by certified pathologists at Virginia Tech on cadavers at 42 days of age.

### MRI

For *ex-vivo* MRI, whole brains were stored in 4% paraformaldehyde (PFA) in 1× Phosphate buffer saline (PBS) for 2 weeks, followed by 1% PFA for 2 weeks, and lastly PBS until use. Forty-eight hours prior to imaging, samples were gently packed with glass beads in cylindrical plastic jars, sealed, filled with Fluorinert^TM^, and incubated on an orbital shaker at 4°C to dislodge any air bubbles trapped in the tissue. Samples underwent MRI on a 9.4 Tesla high field MR instrument (Bruker) at Virginia Tech. T2-weighted images were collected at an in-plane resolution of 125 × 125 μm and slice thinness of 300 μm. Image volumes were analyzed in a double-blinded manner with ITK-SNAP software. Prefrontal cortical white matter and corpus callosum tracts were hand traced slice by slice to obtain volumetrics. An established brain atlas of the domestic pig (Saikali et al., 2010) was used to establish the boundaries of each ROI.

### Free-Floating Fluorescent Immunostaining

Fifty micrometer thick coronal sections were permeabilized in 0.1% Triton-X100 in 1× PBS (PT buffer), blocked with blocking solution (20% normal donkey serum in PT), and incubated at 4°C in primary antibodies diluted in carrier solution (1% BSA, 1% normal donkey serum, 0.3% Triton-X100 in 1× PBS). Next, they were washed with PT buffer (3 × 30 min). Secondary antibody incubation was done in carrier solution for 1.5 h at RT. After washing with PT buffer (3 × 30 min), tissues were mounted on silane- coated slides (TBS- General Data Healthcare, OH). Mounted tissues were covered with DAPI-Fluoromount-G^®^ (Southern Biotech, AL), coverslipped (Fisher Scientific, NH), and light protected and stored at −20°C until imaged.

### Imaging and Analyses

Immunofluorescent images were acquired at 10× and 40× on a Zeiss LSM 880 or Nikon C2 confocal microscope. A minimum of three z-stacks were acquired per animal/group/region of interest with a 2 μm step size. Cell quantification was performed on acquired images using Fiji/ImageJ (NIH) software. To calculate cell densities, the total number of cells counted was divided by the image volume expressed in 10^6^ μm^3^.

### Western Blots

For protein isolation, sliced brain sections were snap-frozen on dry ice directly following brain extraction. Prefrontal cortical white matter and corpus callosum tissues were isolated by micro-dissection in PBS on ice. Protein was extracted using RIPA buffer (Thermo Fisher Scientific, MA) mixed with Halt^TM^ protease and phosphatase inhibitor cocktail (Thermo Fisher Scientific, MA). Protein concentration was determined by a DC protein assay (Bio-Rad, CA). Protein samples were denatured in NuPAGE LDS sample buffer, loaded on NuPAGE 4–12% Bis-Tris gel (ThermoFisher Scientific, MA), and electrophoresis was performed with a Bio-Rad system using 1× MOPS-SDS (Bioworld, OH) as running buffer. Protein transfer was done on Polyvinylidene difluoride (PVDF) membrane using Tris-HCl-based transfer buffer with 20% methanol in a Bio-Rad wet/tank transfer system 4°C. Blots were blocked with 5% milk in 0.1% Tween 20 in 1× Tris-buffered saline (1× TBST buffer). Primary antibodies were diluted in 2% BSA and blots were incubated overnight at 4°C. Horseradish peroxidase (HRP) conjugated secondary antibodies were diluted in 5% milk (1:5,000), and blots were incubated at RT. Clarity western ECL substrate (Bio-Rad, CA) was used for chemiluminescence reaction and blots were imaged on a Bio-Rad chemidoc. Bio-Rad Image Lab software was used to analyze the protein bands and quantify their expression relative to GAPDH or β-Actin bands.

### Antibodies

Antibodies used for immunostaining: rabbit- anti Olig2 (EMD Millipore AB9610, 1:500), mouse-anti APC [CC1] (EMD Millipore OP80, 1:500), mouse-anti Ki67 (BD Pharmingen 550609, 1:50), donkey anti-rabbit IgG (H + L) secondary- Alexa Fluor 568 (Thermo Fisher Scientific A10042, 1:300) and donkey anti-mouse IgG (H + L) secondary- Alexa Fluor 488 (Thermo Fisher Scientific A21202, 1:300). These antibodies have been documented in a previous porcine study (Ishibashi et al., 2012).

Antibodies used for western blots: rabbit-anti MBP (Cell signaling 78896, 1:1,000), rabbit-anti PLP1 (Cell Signaling Technology 85971S, 1:1,000), rabbit-anti HMGCS1 (Cell Signaling Technology 42201S, 1:1,000), rabbit-anti GAPDH (Cell Signaling Technology 5174, 1:1,000), mouse anti-β-Actin C4 (Santa Cruz 47778, 1:1,000), mouse anti-MAG (Santa Cruz 166849, 1:1,000), goat anti-MOG (Novus Biologicals NB300–948, 1:1,000), goat anti-rabbit IgG (H+L)-HRP (Thermo Fisher Scientific G21234, 1:5,000), goat anti-mouse IgG (H+L)-HRP (Thermo Fisher Scientific G16072, 1:5,000), donkey anti-goat IgG (H+L)-HRP (Thermo Fisher Scientific A15999, 1:5,000). Gray matter tissue was used as a negative control.

### Statistical Analyses

All statistical analyses were performed with GraphPad Prism software. Nearly every dataset passed the Shapiro-Wilk normality test to assume a Gaussian distribution. Nonparametric Mann-Whitney U test was performed for the two datasets that did not pass normalcy. Two-way analysis of variance (ANOVA) was done for groups and regions (two or more), whereas a two-tailed unpaired Student’s *t*-test was performed for individual comparisons. Significance was determined if *p*-value was <0.05. Data were expressed in bar graphs as the mean ± standard error of mean (SEM). Significance was shown as *p ≤ 0.05, **p ≤ 0.01, ***p ≤ 0.001. All statistical tests are reported in the figure legends.

## Results

### Characterization of Germ-Free Animals Reveals No Overt Anatomical or Systemic Pathologies

**Figure 1:**
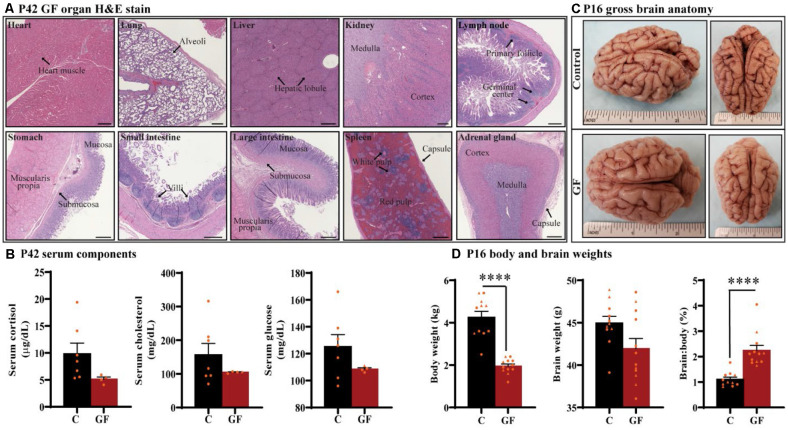
General characterization of germ-free piglets.** (A)** H&E stains of germ-free (GF) organs at postnatal day P42. Scale bars, 500 μm. **(B)** Quantification of cortisol, cholesterol, and glucose levels in the serum of GF and control **(C)** animals at P42. *n* = 4–7 animals/group. **(C)** Gross anatomy of a GF and a control animal at P16. **(D)** Quantification of the total body weight, brain weight, and percent brain to body weight ratio at P16. Individual male and female animals are marked by orange triangles and circles, respectively. *n* = 11–12 animals/group. Data are expressed as mean ± SEM. ****p < 0.0001, unpaired student’s *t*-test for body weight and brain weight, Mann-Whitney U test for percent brain to body weight ratio.

Under the GF paradigm employed in this study, healthy piglets could be housed in sterile isolators up to 6 weeks of age (42 days). To determine if prolonged GF conditions result in any overt pathologies to critical organs that may confound interpretations on brain development, we performed whole-body necropsies at postnatal day 42 (P42). No pathological abnormalities were detected (*n* = 4). Histopathology of vital organs including heart, lung, liver, kidney, and spleen as well as lymph node, stomach, small intestine, large intestine, and adrenal gland showed no irregularities or lesions (Figure 1A). In addition, no intestinal microbes or pathogens were detected in colon isolates.

To determine the impact of sustained GF conditions on peripheral circulatory components that may indicate deleterious internal conditions that could indirectly affect brain development and function, serum from P42 animals was tested for glucose, cholesterol, and the stress-hormone cortisol (*n* = 4–7); no significant differences were detected (Figure 1B). In addition, we found no evidence of excess fat (lipemia), bilirubin (icterus), or hemolysis in GF serum.

Animals were monitored for food intake, communication, lethargy, etc. and no significant differences in behavior were observed in piglets reared in GF isolators. To understand the impact of this environment on brain development in very early life, we assessed brain development on postnatal day 16 (P16; *n* = 11–12). Although brain growth is not linear to age progression, and largely varies among species, P16 piglets are roughly equivalent to 2–3-month-old human infant (Conrad and Johnson, 2015). Body weight was significantly lower in the GF group, which was expected and has been documented in GF rodent models (Bäckhed et al., 2004, 2007); however, no gross anatomical differences or significant changes in brain weight were seen (Figures 1C,D). These changes appeared similar regardless of animal sex, and are even more striking when comparing the ratio of brain to body weight (Figure 1D).

### GF Piglets Display a Significant Reduction in White Matter Volume

**Figure 2:**
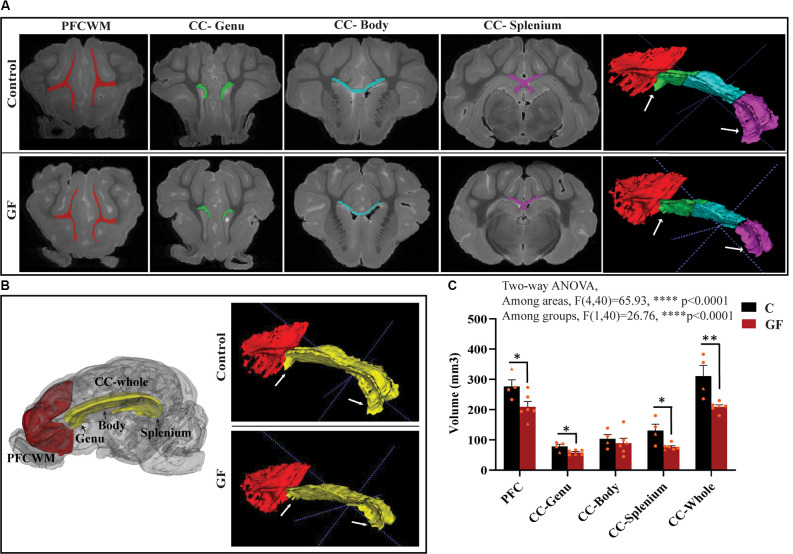
Reduced white matter volume in the prefrontal cortex and corpus callosum in germ-free piglets at 16 days of age. **(A)** Representative coronal planes of MRI images and 3D segmentations illustrating traced regions of interest: PFCWM (red), CC-Genu (green), CC-Body (cyan), and CC-Splenium (magenta). PFCWM, prefrontal subcortical white matter; CC, corpus callosum. **(B)** 3D cartoon of and 3D segmentation of PFCWM (red) and CC-whole (yellow). Black arrows indicate subregions of the CC. White arrows demarcate the CC-genu and CC-splenium. **(C)** Quantification of white matter volumes (mm^3^) in control (C) and germ-free (GF) piglets. Individual male and female animals are marked by orange triangles and circles, respectively. Data expressed as mean ± SEM, *n* = 4–6 animals/group. ****p < 0.0001, two-way ANOVA; *p < 0.05, **p < 0.01, unpaired student’s *t*-test P16-C vs. P16-GF.

In this study, we focused on two key WM tracts in the CNS that are tightly linked to unfavorable behavioral/psychological outcomes in human patients: prefrontal cortical white matter (PFCWM) and corpus callosum (CC). PFC enables higher-order executive function and takes the longest to develop postnatally. On the other hand, CC is the largest commissural WM structure, bundle fibers connecting both hemispheres of the brain, which ensures an earlier developmental timeline compared with PFCWM. Because WM generally develops in a caudal to rostral manner in the brain, we divided the CC into three regions including the genu (most rostral), body, and splenium (most caudal) to account for spatiotemporal differences. A near absence of the characteristic “knee” shape of the genu was noticeable in all GF brains assessed, with smaller curvature at the rear end covering the lateral ventricle (Figure 2A). Quantification of WM volumes revealed a significant reduction in PFCWM, CC-Genu, and CC-Splenium in GF animals compared with controls (Figure 2C). In addition, the volume of the whole CC was significantly less in GF brains (Figures 2B,C). However, there was no significant change seen in total cortical gray matter volume (Supplementary Figure 1).

### GF Piglets Exhibit No Changes in Myelin-Related Protein Density

**Figure 3:**
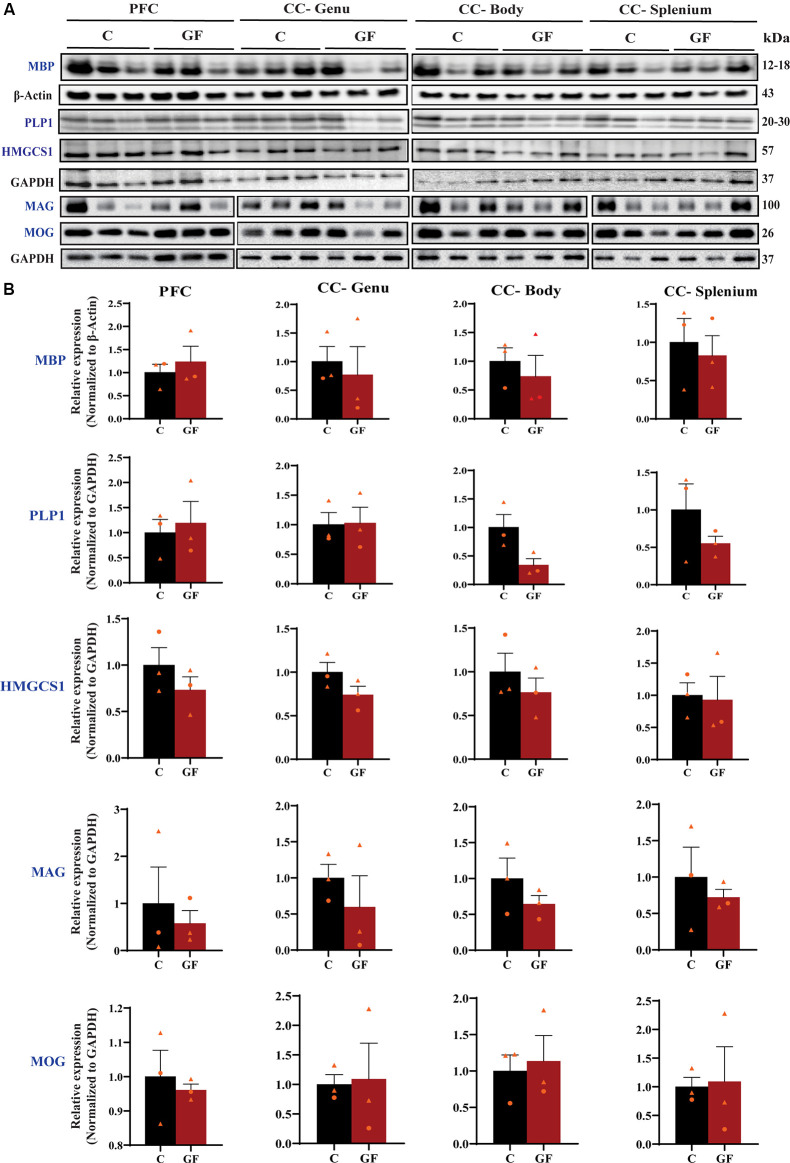
Quantitative assessment of myelin-related protein densities at 16 days of age. **(A)** Western blot analysis of MBP, PLP1, HMGCS1, MAG, and MOG protein (blue font) expression in tissue isolated from the PFCWM, CC-Genu, CC-Body, and CC-Splenium of control (C) and germ-free (GF) animals at P16. Loading controls: β-Actin for MBP, GAPDH for PLP1, HMGCS1, MAG, and MOG. **(B)** Quantification of protein expression normalized to respective loading controls. Individual male and female animals are marked by orange triangles and circles, respectively. Data expressed as mean ± SEM, *n* = 3 animals/group. No significant differences determined by unpaired student’s *t*-test or Mann-Whitney U test. PLP1, proteolipid protein; HMGCS1, Hydroxymethylglutaryl-CoA synthase; MAG, myelin-associated glycoprotein; MOG, myelin oligodendrocyte glycoprotein.

To further explore myelin production, abundance, density, and compaction, we tested the expression of several myelin-related proteins in the PFC and CC. While MBP is an intermembrane protein, proteolipid protein (PLP1) is an intramembrane protein, the most abundant protein in CNS myelin that plays a key role in myelin compaction, stabilization, and maintenance (Lüders et al., 2019). Hydroxymethylglutaryl-CoA synthase (HMG-CoA synthase or HMGCS1) enzyme is necessary for the biosynthesis of cholesterol, which is the main component for myelin production and fluidity of the sheaths. Myelin-associated glycoprotein (MAG) and myelin oligodendrocyte glycoprotein (MOG) are known adhesion proteins important for the structural integrity of myelin sheaths. All these proteins are synthesized by mature myelinating oligodendrocytes. Western blots were performed on proteins isolated from microdissected WM tracts and revealed no significant differences in myelin-associated protein density in GF compared with C animals at P16 (Figure 3). In addition, no sexually dimorphic differences were evident in the limited number of animals assessed. A significant reduction in WM volume detected by MRI (Figure 2), paired with no differences in myelin density (Figure 3), suggests an overall reduction in the total amount of myelin present in each WM tract assessed. Therefore, we next evaluated the presence and abundance of WM-producing cells that occupy WM tracts.

### The Number of Oligodendrocytes and Mature Oligodendrocytes Is Significantly Reduced in GF Piglets

**Figure 4:**
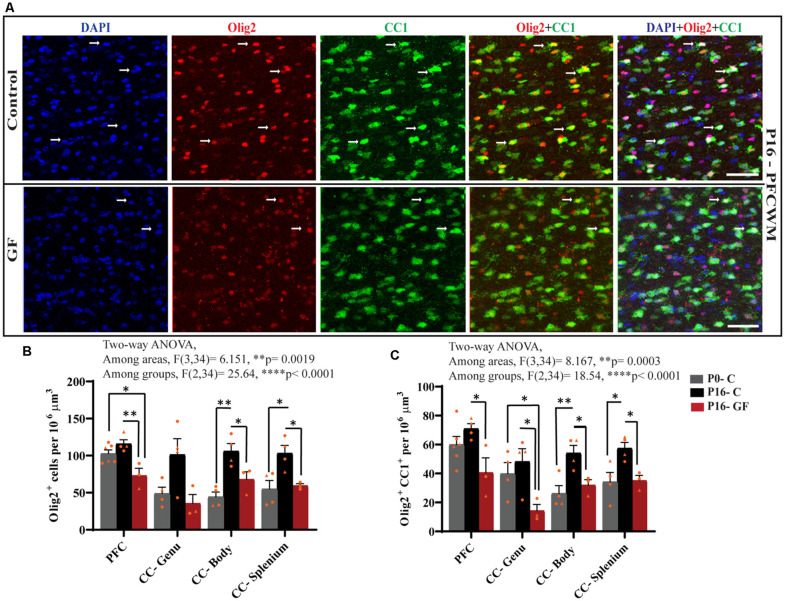
Effects of germ-free conditions on oligodendrocytes and mature oligodendrocytes. **(A)** Representative images showing all oligodendrocytes (Olig2^+^) and mature oligodendrocytes (Olig2^+^CC1^+^) within the PFCWM of control (C) and germ-free (GF) piglets at P16. Scale bar, 50 μm. White arrows demarcate double positive cells. Quantification of the number of Olig2^+^ **(B)** and Olig2^+^CC1^+^ **(C)** cells in PFCWM, CC-Genu, CC-Body, and CC-Splenium in P0-C, P16-C, and P16-GF animals. Individual male and female animals are marked by orange triangles and circles, respectively. Data expressed as mean ± SEM, *n* = 3–6 animals/group. ****p < 0.0001, two-way ANOVA; *p < 0.05, **p < 0.01, unpaired student’s *t*-test.

From oligodendrocyte progenitor cells (OPC) to myelinating oligodendrocytes (OLs), these glial cells undergo complex processes of generation, proliferation, differentiation, maturation, and myelination. In contrast to rodents, pigs and humans are born with mature OLs supporting intact WM tracts, which will continue to undergo growth and refinement postnatally. Therefore, we added another time point to our cell count analyses to serve as a baseline at birth (P0-C). We examined the number of oligodendrocytes per volume in PFCWM and three parts of the CC by immunostaining against Olig2 which is present in all stages of the OL lineage described above. In all areas except the CC-Genu, the number of Olig2+ cells was significantly reduced in the P16 GF group compared with controls (Figure 4B). The number of OLs was significantly higher in the CC-body and splenium in P16 C compared with the P0 baseline, indicating expansion of this cellular pool with age (Figure 4B). We also noted that the number of Olig2+ cells in P16 GF animals was even lower than P0 in PFC and CC-Genu; interestingly, this observation was more severe and significant in the least mature WM tract assessed, the PFC, suggesting there may be an impairment in OL production or enhanced cell pruning (Figures 4A,B).

To determine any effects of GF conditions on OL maturation that may impact myelination and total WM volume, we immunolabeled coronal tissues with CC1 and Olig2. Along with Olig2, cells double-positive for Olig2 and CC1 allowed us to quantify the mature OL population per volume within these WM tracts. In all regions assessed, at P16 the number of mature OLs was significantly reduced in the GF animals compared with controls. Similar to the number of Olig2+ cells, the number of mature OLs in PFC and CC-Genu was even lower than P0, with a significant reduction in the CC-Genu (Figure 4C). In addition, there was a significant age-related increase in mature OLs from P0 to P16 with the CC-Body and splenium of control animals. Overall, these findings may be indicative of a loss of mature OLs, reduced oligodendrogenesis, or delays/arrest in OL maturation (Figures 4A,C). Male animals seemed to be more affected both in the Olig2 and Olig2+CC1 populations.

### No Changes in Oligodendrocyte Proliferation Within the SVZ of GF Piglets

**Figure 5:**
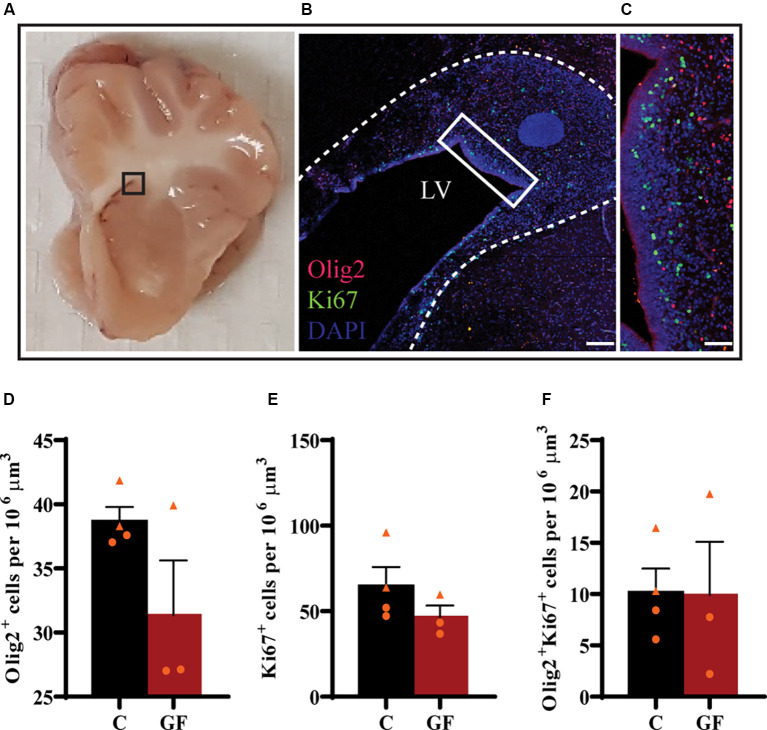
No effects of germ-free conditions on oligodendrogenesis within the subventricular zone at P16.** (A)** Gross anatomical map to demarcate region of interest **(B)**. **(B)** Low magnification image of the SVZ, immunolabeled with Olig2 and Ki67, corresponding to inset (in **A**). **(C)** Higher magnification image of inset (in **B**). Quantification of the number of oligodendrocytes **(D)**, proliferating cells **(E)**, and proliferating oligodendrocytes **(F)**. LV, lateral ventricle; C, control; GF, germ-free. Data not significant by unpaired student’s *t* test. Data expressed as mean ± SEM, *n* = 3–4 animals/group.

Upon the observation of lower OL and mature OL numbers, we evaluated the proliferation capacity of OPCs. The subventricular zone (SVZ) represents a major source of newborn OLs (Menn et al., 2006). From the SVZ OPCs migrate to different target sites in the brain, proliferate and differentiate into mature myelinating OLs. Some OPCs remain in local pools in these WM sites where they can proliferate, differentiate, and myelinate throughout a lifetime. We examined the proliferation of OPCs in the SVZ by immunostaining against Olig2 and Ki67 (Figures 5A–C). Ki67 is a widely used nuclear marker to identify proliferating cells. There were no significant changes in the number of Olig2+ cells (Figure 5D), proliferating cells (Figure 5E), or proliferating OPCs (Figure 5F) per volume between control and GF groups at P16.

### Local Oligodendrocyte Proliferation Is Significantly Reduced in Early Postnatal GF Conditions

**Figure 6:**
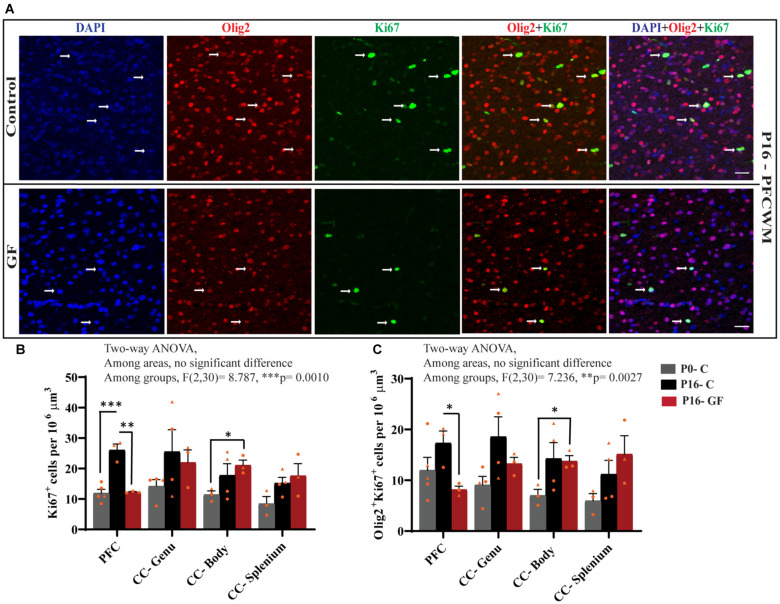
Region-specific reduction oligodendrocyte proliferation in germ-free piglets at P16. **(A)** Representative confocal images showing all cells (DAPI+, blue), OLs (Olig2+, red), proliferating cells (Ki67+, green), and proliferating OLs (Olig2+Ki67+, yellow) and merged images in P16 control and germ-free (GF) animals. Scale bars, 50 μm. Quantification of the number of Ki67+ cells **(B)** and Olig2+Ki67+ proliferating OLs **(C)**. Individual male animals are marked by yellow triangles and female animals are marked by yellow circles. Data expressed as mean ± SEM, *n* = 3–5 animals/group. **p < 0.0001, two-way ANOVA; *p < 0.05, **p < 0.01, ***p < 0.001, unpaired student’s *t*-test.

In accordance with the findings that OL proliferation is not affected by GF conditions in the SVZ, but OL numbers and OL maturation are significantly lower in PFC, CC-Genu, CC-Body, and CC-Splenium, we considered the underlying cause may be a reduction in local pools of OPCs within these WM tracts. In agreement with our hypothesis, the number of Ki67+ proliferating cells per volume was significantly lower in PFCWM in the P16-GF compared with P16-C group, but not in any region of the CC (Figures 6A,B). In PFCWM, there was no significant difference between P0-C and P16-GF animals, indicating a halt or delay in cell proliferation in this area at the front of the brain (Figures 6A,B). A significant reduction in proliferating OLs (Olig2+ Ki67+) was seen in PFCWM of GF piglets compared with controls (Figures 6A,C). These findings indicate a halt in cell proliferation in PFCWM at birth, when the gut is initially colonized with microbiota, resulting in less mature OLs to build upon key WM tracts. Similar to the observations in mature OL in PFC, male animals appeared to be more affected.

## Discussion

The influence of microorganisms on brain development has recently been brought into the clinical and research forefront; however, almost nothing is known regarding the impact of microbiota on WM development. Here, we utilized a germ-free swine model, completely absent of microbiota, and report findings revealing changes in key WM tracts related to the numerous neurological diseases/disorders commonly associated with gut dysbiosis. The complete absence of all microorganisms eliminates confounding variables such as antibiotic-resistant bacteria (Kennedy et al., 2018). Antibiotic depletion can also affect other cell types such as microglia and inadvertently damage mitochondria, which are crucial for the proper development of stem cell niches giving rise to OLs (Zhang et al., 2005; Beckervordersandforth et al., 2017; Fernandez et al., 2019). Owing to the precocial nature of newborn pigs, our model is endowed with the advantage of a controlled diet obviating confounding variables obtained from other animal models where neonates nurse from GF or antibiotic depleted dams. Histopathological assessments revealed no overt pathologies throughout the body or serum, strengthening the interpretation of our findings as resultants of an absence of microorganisms vs. damage to other vital organs that may have an impact on brain development (Figure 1). Previous studies have shown that GF piglets show reduced weight of specific organs including heart, lung, liver, spleen, and kidney (Zhou et al., 2021) which may account for, in part, the reduced body weights seen (Figure 1).

**Figure 7:**
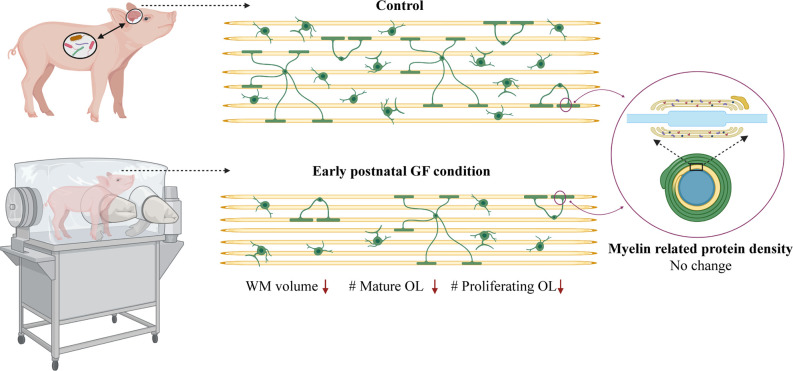
Data summary. WM volume, number of mature oligodendrocytes and number of proliferating oligodendrocytes is reduced in select WM tracts (PFC and CC) in early postnatal GF condition compared to control piglets. No change was observed in the expression of myelin related proteins in the GF condition. GF, germ-free; WM, white matter; OL, oligodendrocyte. Figure created with BioRender.com.

Here, we demonstrate aberrations in WM development including, reduced WM volume (Figure 2), and a reduction in OPC proliferation locally within distinct WM tracts (Figure 6) paired with fewer mature OLs (Figure 4) in a gyrencephalic mammal that is near bioequivalent to humans (summarized in Figure 7). We found no reduction in myelin-associated proteins; however, our analyses were performed on proteins isolated directly from microdissected WM tracts and only reflect density. Considering a loss of WM volume determined macroscopically by MRI (Figure 2), these findings suggest an overall reduction in the total amount of myelin which seems to contradict the hypermyelination found in the PFC of GF mice (Hoban et al., 2016). It is important to note that the aforementioned study made evaluations at 10 weeks of age, whereas our findings are during early postnatal life. Future studies employing other techniques such as electron microscopy are necessary to confirm this possibility as well as lend insights into possible changes in axonal numbers, diameter, myelin thickness, and compaction which were not evaluated in the present study. In addition, because WM tracts are also occupied by a diversity of cells including WM astrocytes, interstitial neurons, and endothelial cells, it will also be important to evaluate potential aberrations in these cellular populations in germ-free piglets. In addition, although we found no significant differences in cortical volume by MRI, we cannot rule out the possibility that there are differences in cell numbers or dendritic arborization in the cortex of GF piglets.

At birth, microorganisms derived from the mother and the surrounding environment colonize the newborn gut, while WM continues to develop in concert. Diffusion Tensor Imaging (DTI) studies have shown that by 24–32 gestational weeks major WM tracts, such as the corpus callosum (CC), are easily identifiable, while cortical WM tracts lag behind (Qiu et al., 2015). Although the present study only evaluated changes in WM development at age 1-month, which is the human equivalence of a 2–3-month-old infant (Conrad and Johnson, 2015), there is information to be gleaned regarding temporal aspects of development because myelination is an asymmetric process. Compared with the CC, complete myelination of the WM tracts underlying the PFC takes considerably longer; therefore, we considered the PFCWM to be less mature during the epoch of brain development studied. In addition, WM maturation within the CC is a variable process spanning the genu (rostral), body (middle), and splenium (caudal; Luders et al., 2010). We found the most drastic differences in the PFCWM and the genu of the CC, suggesting higher vulnerability in the less mature WM tracts residing near the front of the brain. In humans, structural components of the CC develop along the midline during very early gestation and subsequently spread to rostral and caudal ends (Jovanov-Milosević et al., 2009). This may be reflected in our WM volume findings, showing significant reductions in the CC-genu and CC-Splenium, but no change in the CC-Body (Figure 2). Roughly, WM fibers from two parts of the frontal lobe (executive functions, cognition, etc.) form the genu, while the body is formed by fibers belonging to the parietal lobe (motor functions), and the splenium mostly consists of the fibers of the temporal and occipital lobes (visuo-spatial functions, memory, emotion, etc.; Hofer and Frahm, 2006). It will be interesting to see if these spatial changes in specific WM tracts are predictive of behavioral deficits related to their respective functions; for example, alterations in higher-order functions (e.g., executive and cognitive) with no impact on motor functions.

OPCs represent an abundant cellular pool responsive to environmental stressors following birth, they are capable of generating mature oligodendrocytes that can myelinate up to 100 axons and potentially serve as master synchronizers of neural circuit activity. Therefore, even a small loss in OLs can have drastically negative effects on mental health (Xin and Chan, 2020). While we found a significant reduction in the number of mature OLs (Figure 4), we cannot rule out the possibility that OL cell death and/or delayed maturation partially account for this loss. However, the reduction in OL proliferation (Figure 6) suggests that delayed or impaired oligodendrogenesis is a key factor resulting in fewer mature OLs in GF piglets. A potential underlying molecular mechanism for this phenotype may involve TGF-β signaling and its link to oligodendrogenesis and WM development. It has been reported that GF mice show significantly reduced gene expression of TGF-β in the gut (Bauché and Marie, 2017). In addition, TGF-β signaling is known to promote OPC differentiation and maturation (Palazuelos et al., 2014). Therefore, a reduction in TGF-β levels in the periphery may impact WM development in GF piglets. Future studies evaluating whether this is a transient or sustained phenomenon responsive to interventions such as gut recolonization will be essential in understanding the cellular mechanisms at play.

GF animals are derived and maintained free from microorganisms including bacteria, viruses, parasites, and fungi. Comparative studies in antibiotic-treated and GF mice have shown that GF animals are not only devoid of microorganisms in the gut, but also in other microbiome niches. GF piglets are also deprived and lack early immune education which may impact overall cell signaling pathways during development (Kennedy et al., 2018). Although most studies ascribe alterations in brain development to the gut microbiota, there are other niches in the vagina, skin, and breast (Backhed et al., 2015; Sharon et al., 2016; Vuong et al., 2017). In order to establish a more causal link between gut microbiota and the WM phenotypes documented in this study, it will be essential to recolonize the gut with healthy swine gut microbiota. In addition, it is of great importance to determine if the influence of the gut microbiome is due to developmental or actively modulated processes; as recolonization later in life or near adolescence often results in mixed outcomes even within the same study. Future studies employing such recolonization paradigms will aid in determining optimal windows for therapeutic interventions *via* minimally invasive strategies.

There are some limitations in studying the gut microbiome in the pig model that are noteworthy. Pigs have a bigger colon as a percentage of the whole gastrointestinal tract compared to humans. Therefore, the estimated contribution of SCFAs to their basal metabolic requirement is high in pigs (30–76%) and only 5–9% in humans and rodents (Sciascia et al., 2016). Another limitation specific to our model is how quickly GF piglets outgrow the size of the isolators, restricting our analyses to 6 weeks of age; however, future studies utilizing larger isolators designed for cattle will aid in overcoming this issue. In addition, pigs possess an epitheliochorial placenta (three layers of tissue separating the fetus from maternal blood) in contrast to a hemochorial placenta (fetal chorionic epithelium is bathed in maternal blood) in humans and rodents (Furukawa et al., 2014). As a result, IgGs do not pass to the fetus during gestation; instead, they receive this *via* colostrum after birth (Borghesi et al., 2014). Although derivation *via* hysterectomy ensures the complete absence of microbiota (GF condition) it is important to keep these issues in mind when making comparisons to humans.

Piglet brain growth mirrors sexual dimorphisms seen in humans (Conrad et al., 2012) which is an important variable in WM composition as males are known to have more WM volume than females in adulthood (Allen et al., 2003; Bourisly et al., 2017). In our study with a limited number of animals, we could not perform statistical analyses to determine significant sex-specific differences. However, males seemed to be more vulnerable to GF conditions with more severe reductions in mature and proliferating OLs (Figures 4, 6). Among the diseases associated with alterations in the gut microbiome and WM, autism spectrum disorder (ASD) is more prevalent in males with a 4–1 ratio (Werling and Geschwind, 2013). In addition, the age of onset for schizophrenia typically occurs earlier in males than females (Li et al., 2016). Sex differences are also seen in attention-deficit hyperactivity syndrome (ADHD), with higher incidences in males which vary among age and disease subtypes (Ramtekkar et al., 2010). Importantly, these diseases are associated with gut dysbiosis, whereas our experimental paradigm reflects changes owing to a complete absence of microorganisms. Future studies with larger cohorts of animals will be important to determine if there are sex-specific differences in WM development in the absence of microbiota in piglets.

In conclusion, we established a postnatal swine model to study the effects of microbiota on WM development including volumetric, cellular, and biochemical analyses. Our GF study corroborated with the spatio-temporal profile of WM maturation with aberrations prominent in the most frontal WM tracts of the brain whose maturation occurs primarily from postnatal through adolescence periods. These findings, along with the aforementioned lines of inquiry to be addressed in the future, will aid in designing clinical strategies such as pre-, pro-, and symbiotics to improve mental health in neonatal, pediatric, adolescent, and even adult populations suffering from a broad array of neurological deficits associated with gastrointestinal comorbidities.

## Data Availability Statement

The original contributions presented in the study are included in the article/Supplementary Material, further inquiries can be directed to the corresponding author.

## Ethics Statement

The animal study was reviewed and approved by Institutional Animal Care and Use Committees (IACUC).

## Author Contributions

PDM designed the experiments and conceived the project. MW, SL, and BRH optimized MRI scans. SA and JM performed MRI analyses. AP, SH, and HD performed western blots. SA quantified and analyzed Western blot, performed statistical analyses, prepared the figures, and wrote the manuscript with PDM. SA, ST, FD-B, AR, and JJ performed free-floating immunohistochemistry. SH, AP, and PDM performed confocal microscopy. SA, ST, FD-B, AR, AB, and JJ analyzed confocal images. PDM, LY, SA, DP, SH, and AKR assisted in sow hysterectomies, germ-free piglet rearing, sterility checks, blood draws, and euthanasia. PDM, SA, DP, and SH contributed to brain extraction. SH processed brain tissue. SA and PDM analyzed experimental data. All authors contributed to the article and approved the submitted version.

## Conflict of Interest

The authors declare that the research was conducted in the absence of any commercial or financial relationships that could be construed as a potential conflict of interest.

## Publisher’s Note

All claims expressed in this article are solely those of the authors and do not necessarily represent those of their affiliated organizations, or those of the publisher, the editors and the reviewers. Any product that may be evaluated in this article, or claim that may be made by its manufacturer, is not guaranteed or endorsed by the publisher.

## Supplementary Materials

Supplementary Figure 1 Assessment of cortical gray matter volume at 16 days of age. **(A)** Representative axial plane of MRI image and 3D segmentations illustrating total cortical gray matter. **(B)** Quantification of total cortical gray matter volume (mm^3^) in control **(C)** and germ-free (GF) piglets. Individual male and female animals are marked by orange triangles and circles, respectively. Data expressed as mean ± SEM, *n* = 4–6 animals/group. No significant differences determined by unpaired student’s *t*-test.
