# Supplementary figures and images for: Early Influences of Microbiota on White Matter Development in Germ-Free Piglets
Source: Front Cell Neurosci. 2021 Dec 27;15:807170. doi: 10.3389/fncel.2021.807170 (PMC8751630; doi:10.3389/fncel.2021.807170)

**Supplementary Figure 1**

**
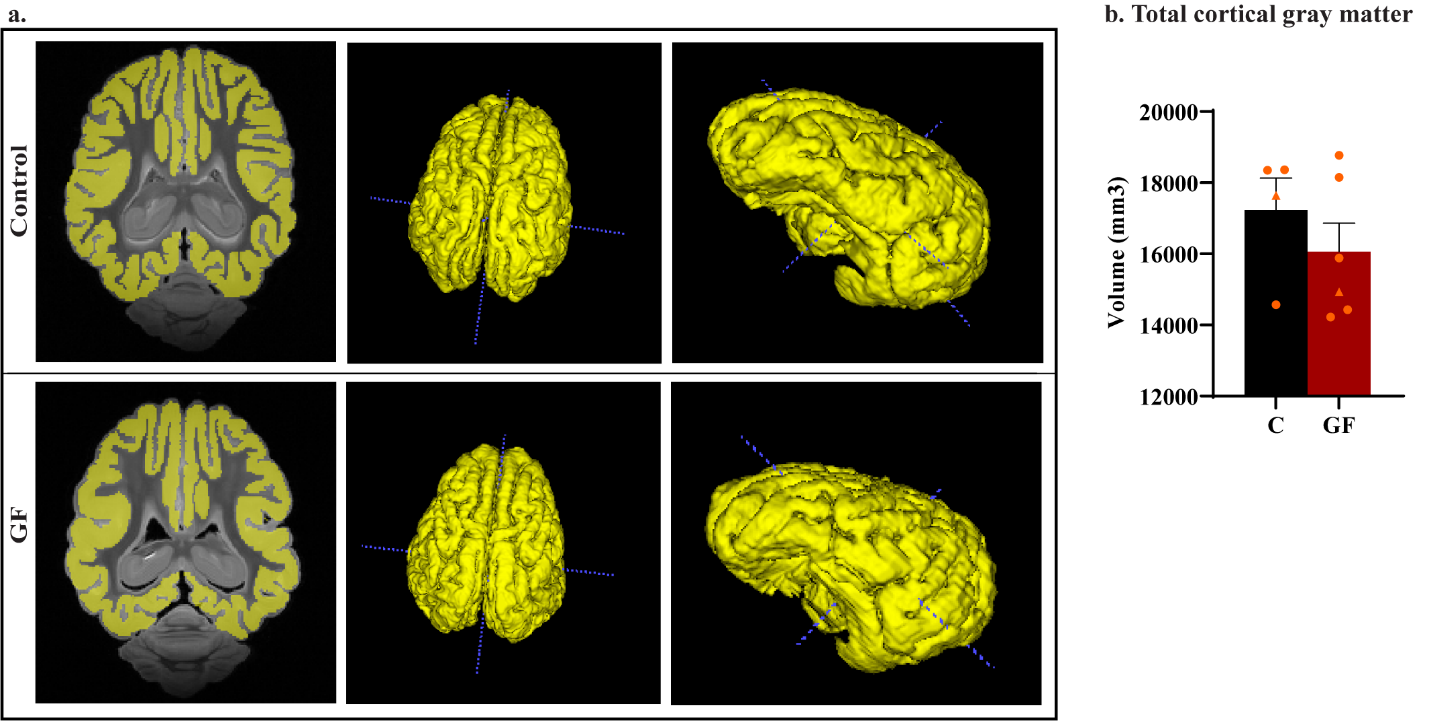
**

Supplement: Supplementary Figure 1 — Assessment of cortical gray matter volume at 16 days of age. (A) Representative axial plane of MRI image and 3D segmentations illustrating total cortical gray matter. (B) Quantification of total cortical gray matter volume (mm3) in control (C) and germ-free (GF) piglets. Individual male and female animals are marked by orange triangles and circles, respectively. Data expressed as mean ± SEM, n = 4–6 animals/group. No significant differences determined by unpaired student’s t-test. [file Data_Sheet_1.docx]
